# UBAP2L silencing inhibits cell proliferation and G2/M phase transition in breast cancer

**DOI:** 10.1007/s12282-017-0820-x

**Published:** 2017-12-01

**Authors:** Jing He, Yuanping Chen, Lu Cai, Zelei Li, Xiaoqing Guo

**Affiliations:** Department of Oncology, The Affiliated Ganzhou Hospital of Nanchang University, No. 17 Hongqi Avenue, Ganzhou, 341000 Jiangxi China

**Keywords:** Breast cancer, CDK1, Cell proliferation, Cyclin B1, G2/M phase, UBAP2L

## Abstract

**Background:**

Ubiquitin-associated protein 2-like (UBAP2L) contains a ubiquitin-associated domain near its N-terminus, which has been demonstrated to be overexpressed in multiple tumors, including hepatocellular carcinoma and colorectal carcinoma but its role has not been well studied in breast cancer. Thus, this study was designed to evaluate whether UBAP2L can serve as a potential molecular target for breast cancer therapy.

**Methods:**

The expression of UBAP2L was determined in breast cancer tissues and cell lines by Western blotting and Oncomine database mining. Then the expression of UBAP2L was silenced using RNA interference and the effects of UBAP2L knockdown on breast cancer cell proliferation and cell cycle progression by MTT and colony formation assay, and Flow cytometry, respectively.

**Results:**

We found the expression of UBAP2L was significantly up-regulated in breast cancer tissues and cell lines. Knockdown of UBAP2L suppressed cell proliferation, impaired colony formation ability and induced cell cycle arrest at G2/M phase. At molecular levels, knockdown of UBAP2L increased p21 expression, but decreased the expression of CDK1 and Cyclin B1 in breast cancer cells.

**Conclusion:**

Our findings suggest that UBAP2L plays an important role in breast cancer cell proliferation and might serve as a potential target for breast cancer treatment.

## Introduction

Breast cancer is the main malignant tumor among women, accounting for about 33% of cancer cases in the United States (US) [1], which has been the second leading cause of death for women, nearly 40,000 deaths (US) [1]. Many breast cancer patients presented distinct metastasis, including lymph nodes, lung liver, and bone marrow [2]. Although technological advances in the diagnostic and treatment, the risk of mortality in metastatic breast cancer remains high due to limitations of effective strategies [3, 4]. There is an urgent need to develop new therapeutic alternatives for fighting against this disease. A majority of genes are reported to be involved in progression and metastasis of breast cancer [5]. Therefore, identification of such factors may provide a potential target for gene therapeutic in breast cancer.

The ubiquitin-proteasome system (UPS) is a major mechanism for cellular protein degradation in eukaryotic cells [6]. Besides, ubiquitin-like proteins modifications and proteins that constitute the ubiquitin element can influence diverse physiological and pathological processes [7]. Ubiquitin-associated protein 2-like (UBAP2L) is a highly conserved protein that contains an N-terminal ubiquitin-associated (UBA) domain RGG/RG repeats [8] and considered to participate in UPS and the aggregate formation induced by proteasome inhibitor [9]. It can interact with BIM1 a Polycomb group (PcG) protein to form a complex and modulate the hematopoietic stem cells activity [10]. Lingerer, a *Drosophila* ortholog of the human UBAP2L gene, could regulate growing tissues proliferation though modulating JAK/STAT signaling pathway [11]. Recent studies have confirmed that UBAP2L function as an oncogene and is associated with various types of cancer, including prostate cancer [12], glioma [13], hepatocellular carcinoma (HCC) [14], and colorectal carcinoma [15]. However, the effect of UBAP2L expression on breast cancer biology remains largely uncovered.

In this study, the expression of UBAP2L was determined in breast cancer tissues and cells. We also investigated the biological characteristics in tumor cells by loss-of-functional assay and demonstrated that UBAP2L knockdown led to decreased cell proliferation, impaired colony formation and arrested cell cycle.

## Materials and methods

### Clinical tissue specimen collection

Total eight breast cancer tissue and pair-matched paracarcinoma tissue specimens were provided by the Department of Oncology, The Affiliated Ganzhou Hospital of Nanchang University from December 2015 to October 2016. Of these eight samples, three were Luminal B (ER+/PR+, HER2−), two were Basal-like/triple negative (ER−,PR−, HER2−) and the remaining three were Luminal A (ER+/PR+, HER2+) based on the previous report [16]. All samples were obtained surgically and immediately snap frozen in liquid nitrogen and stored at − 80 °C before use. The tissue procurement protocol used in the study was approved by the Institutional Review Board of the Affiliated Ganzhou Hospital of Nanchang University (Jiangxi, China). All patients were required to sign the appropriate informed consent before enrollment.

### Cell lines and culture

Breast cancer cell lines, MCF-7 (ER+/PR+), ZR-75-30 (ER+, HER2+), BT-474 (ER+, HER2+), T-47D (ER+/PR+) and MDA-MB-468 (ER−,PR−, HER2−) and one normal breast epithelial cell line, MCF-10A (used as a negative control), were obtained from the Cell Bank of Chinese Academy of Sciences (Shanghai, China). MCF-7 and T-47D cells were cultured in DMEM (Hyclone) containing 10% fetal bovine serum (FBS, Gibco, USA). MCF10A were cultured in DMEM/F12 medium supplemented with 10% FBS. MDA-MB-468 were cultured in L15 containing 10% FBS. ZR-75-30 and BT-474 cells were cultured in RPMI-1640 (Hyclone) medium supplemented with 10% FBS. All cell lines were maintained at a temperature of 37 °C in atmosphere of 5% CO_2_.

### Lentivirus construction and cell transfection

Total of three shRNA sequences targeting human UBAP2L (NM_001127320.1) gene, including shUBAP2L-1, shUBAP2L-2 and shUBAP2L-3 and the negative control shRNA sequence (NC) were designed and described in Table 1. Lentivirus construction of shUBAP2L and mock-shRNA were purchased from Shanghai Genechem Company. For cell transfection, ZR-75-30 and T-47D were seeded into six-well plates and transfected with lentiviral particles, including shUBAP2L-1, shUBAP2L-2 and shUBAP2L-3 and NC, respectively. The cells without transfection were used as blank control group. At 96 h after transfection, cells were harvested to verify UBAP2L silencing efficiency using real-time PCR and Western blotting.

**Table 1:** The sequences of shRNA used in this study

shRNA	shRNA sequence (5′–3′)
shCon	CCGGTTCTCCGAACGTGTCACGTTTCAAGAGAACGTGACACGTTCGGAGAATTTTTG
shUBAP2L-1	CACCGCATGTTAGGGAAAGGATTTGCGAACAAATCCTTTCCCTAACATGC
shUBAP2L-2	CACCGCCAGCCAATACTGATGATAACGAATTATCATCAGTATTGGCTGGC
shUBAP2L-3	CACCGGGAAGACACCATCTACAATGCGAACATTGTAGATGGTGTCTTCCC

### Quantitative real-time PCR

Total RNA from samples or cells was isolated using Trizol reagent (Gibco) and the first-strand cDNA was generated using Oligo (dT) primer and the M-MLV Reverse Transcriptase (Promega) according to manufacturer’s instructions. Quantitative real-time PCR was conducted using CFX Connect™ Real-Time PCR Detection System (Takara). The mixture, including 10 µl 2 × SYBR premix ex-taq, 0.5 µl primer, 5 µl cDNA and 4.5 µl ddH_2_O, were used for quantitative real-time PCR. The primer sequences used were: UBAP2L (forward): 5′-ACACAATCCCCATCACTGGT-3′, UBAP2L (reverse): 5′-CAGAGGAGAAGACGGAGGTG-3′; β-actin (forward): 5′-GTGGACATCCGCAAAGAC-3′, β-actin (reverse): 5′-AAAGGGTGTAACGCAACTA-3′. The quantification analysis was performed by the $2^{{ - \Delta \Delta C_{\text{t}} }}$ method [17]. The β-actin mRNA was used for normalization. The experiment was performed in triplicate and repeated three times.

### Western blotting

Protein obtained from samples or cells was extracted using Total Protein Extraction Kit following the manufacturer’s instructions. Protein quantification was carried out using BCA protein assay kit. After quantification, total proteins were separated in 10% SDS-PAGE gel electrophoresis and transferred onto PVDF membranes. Next, the transferred PVDF membranes were treated with a blocking buffer (5% non-fat milk dissolved in TBST solution) for 1 h at room temperature. Then, the membrane was incubated overnight at 4 °C with the following antibodies: anti-UBAP2L (ab70319, Abcam), CDK1 (19532-1-AP, Proteintech), CyclinB1 (#21540, SAB), P21 (#2947, Cell Signaling) and GAPDH (#10494-1-AP, Proteintech). The membranes were washed three times with TBST and then incubated with horseradish peroxidase-conjugated goat anti-rabbit IgG antibody (#Sc-2054, Santa Cruz) for 1 h at room temperature. Finally, the immunoreactive bands were detected using enhanced chemiluminescence. GAPDH protein was used as an internal control.

### MTT and colony formation assays

For MTT assay, transfected cells were reseeded in a sterile 96-well plate at a density of 2500 cells/well and grew for consecutive 5 days. Total 20 µl of 5 mg/ml MTT (Sigma-Aldrich) were added to each well and incubated with cells for 4 h in the incubator. Then the medium was removed and 100 µl of DMSO was added into each well to dissolve the formazan. Finally, the optical value (OD) in each well was read using a spectrophotometer at a wavelength of 595 nm. The experiment was performed in triplicate and repeated three times.

For colony formation assay, transfected cells were reseeded into six-well plate with 500 cells per well. After approximately 5 days of cultivation, the natural colonies were washed with 1 × PBS and fixed with 4% paraformaldehyde for 30 min at room temperature. Then the colonies were stained with crystal violet for approximately 20 min. Finally, the stained colonies were imaged and quantified. The experiment was performed in triplicate and repeated three times.

### Cell cycle analysis

After 96-h transfection, cells were reseeded in 6-cm dishes at a density of 100,000 cells per dish. Until cells reached 80% confluency, cells were collected re-suspended in cold PBS, and fixed in 75% ethanol overnight. Then fixed cells were washed with PBS and collected by centrifugation. Subsequently, cells were incubated with PBS containing 50 µg/ml Propidium iodide (PI) and RNase (Sigma-Aldrich) at 37 °C for 1 h. Finally, Cell cycle distribution was analyzed by flow cytometer (BD Biosciences, USA) equipped with *ModFit* LT DNA analysis program. The experiment was performed in triplicate and repeated three times.

### Bioinformatics meta-analysis

To widely investigate the expression profile of UBAP2L in breast cancer tissue vs. normal breast tissue, we conducted a meta-analysis on online microarray data in the Oncomine database (http://www.onocomine.org) by setting the following search terms: “UBAP2L”, “Cancer vs. Normal Analysis”, “Breast Cancer” and “mRNA”. All data are reported Log2 Median-Centered intensity in the Oncomine database.

### Statistical analysis

All results were drafted in diagrams by GraphPad Prism 5 software and all quantitative data were expressed as mean ± standard deviation (SD) of at least triplicate determination. The Student’s *t* test was used to evaluate the continuous variables of two groups (blank vs. NC or NC vs. shUBAP2L) using SPSS version 19 (IBM Corporation, USA). *P* value < 0.05 was considered statistically significant.

## Results

### UBAP2L was upregulated in breast cancer tissues and cell lines

**Fig. 1:**
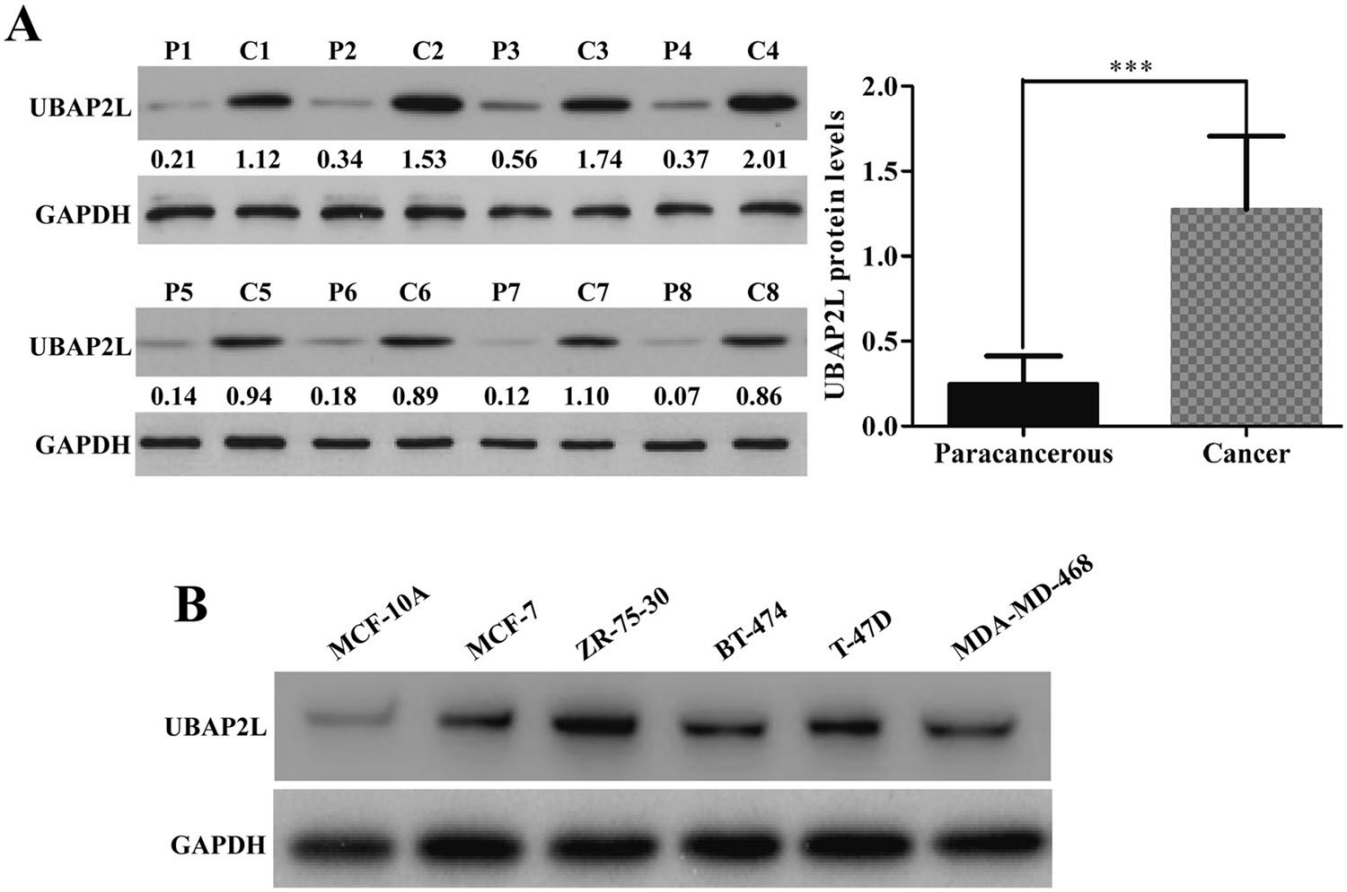
UBAP2L expression is frequently upregulated in breast cancer tissues and cell lines. **a** Western blotting analysis was performed in three cases of fresh breast tissues and paired paracarcinoma tissues. *P* paracarcinoma tissues, *C* cancer tissues; **b** MCF-7, ZR-75-30, BT-474, T-47D, MDA-MB-468 and MCF-10A cell lines were used to analyze UBAP2L expression using Western blotting analysis. GAPDH was used as internal control. ***P < 0.001 vs. precarcerous tissues

The UBAP2L expression was analyzed in eight pairs of fresh breast cancer tissues and paired paracarcinoma tissues by Western blot. As shown in Fig. 1a, the UBAP2L expression level in breast cancer tissue was significantly upregulated than that in the paracarcinoma tissues (*P* < 0.001). Consistent with the findings in breast cancer tissues, the expression of UBAP2L protein was upregulated in breast cancer cell lines, including MCF-7, ZR-75-30, BT-474, T-47D and MDA-MB-468 compared with normal breast cell lines, MCF-10A (Fig. 1b). These results suggest that UBAP2L expression is frequently upregulated in human breast cancer. Notably, ZR-75-30 and T-47D cells exhibited relatively higher UBAP2L expression among the five breast cancer cell lines and thus were chosen for loss-of-function study.

### Knockdown of UBAP2L in breast cancer cells through lentivirus-mediated expression of shRNA

**Fig. 2:**
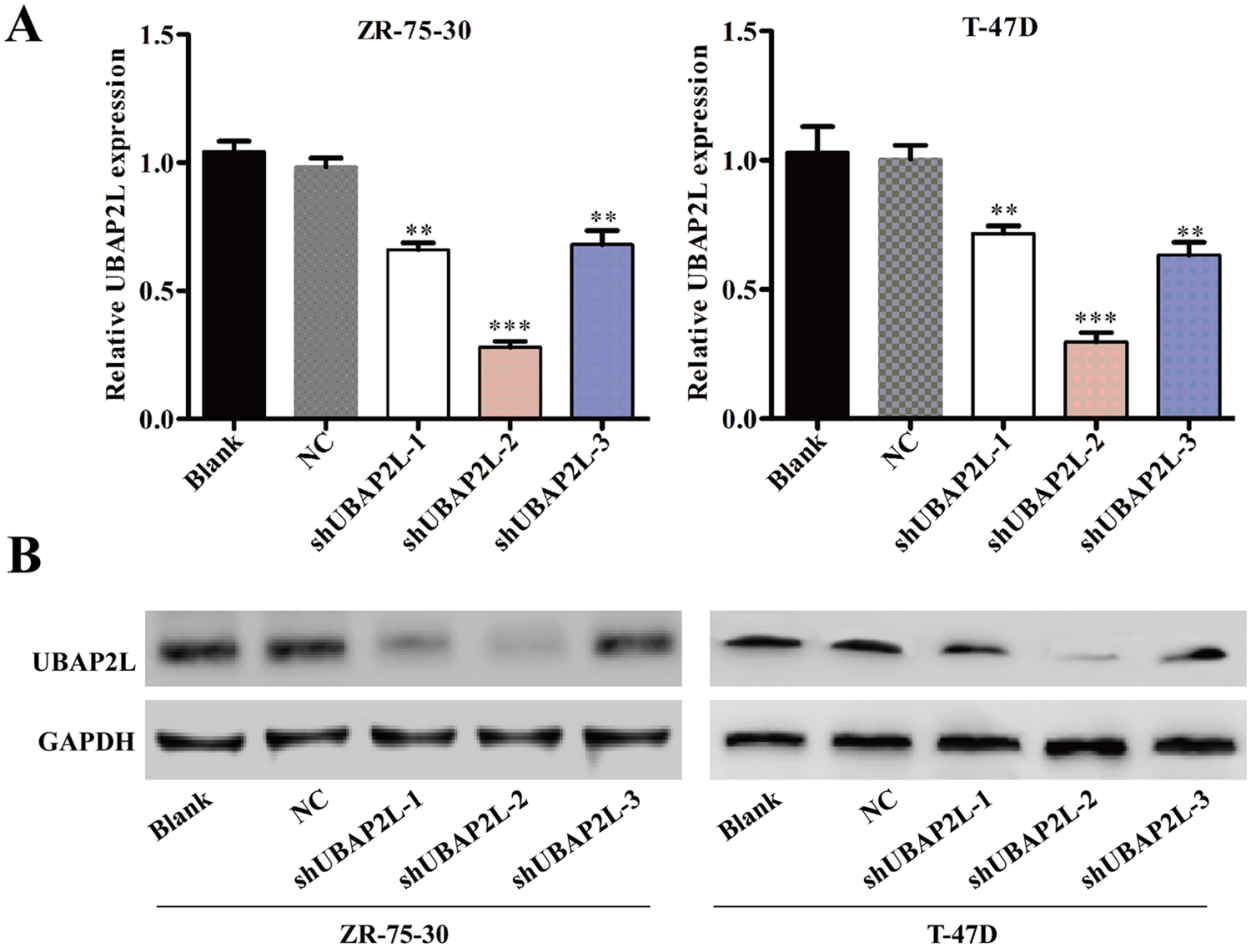
Determination of silencing efficiency of lentivirus-mediated RNA interference against UBAP2L in breast cancer cells. Knockdown of UBAP2L by shRNA was confirmed by **a** quantitative real-time PCR and **b** Western blotting analysis. Data represent the mean ± SD of triplicate samples. GAPDH was used as internal control. **P < 0.01, ***P < 0.001 vs. negative control (NC)

To further explore the biological significance of UBAP2L in breast cancer, lentivirus-introduced shRNA was used to silence UBAP2L expression in ZR-75-30 and T-47D cells. The efficacy of UBAP2L downregulation was confirmed by quantitative real-time PCR and Western blot analyses. As shown in Fig. 2a, shUBAP2L-2 resulted in nearly 80% downregulation of UBAP2L mRNA expression (*P* < 0.001), while shUBAP2L-1 and shUBAP2L-3 resulted in about 30% of that (*P* < 0.01) in both ZR-75-30 and T-47D cells. Similarly, shUBAP2L-2 caused more remarkable downregulation of UBAP2L protein expression than that in shUBAP2L-1 and shUBAP2L-3 (Fig. 2b). Therefore, shUBAP2L-2 was chosen to further studying.

### Knockdown of UBAP2L impaired breast cancer cell proliferation and colony formation ability

**Fig. 3:**
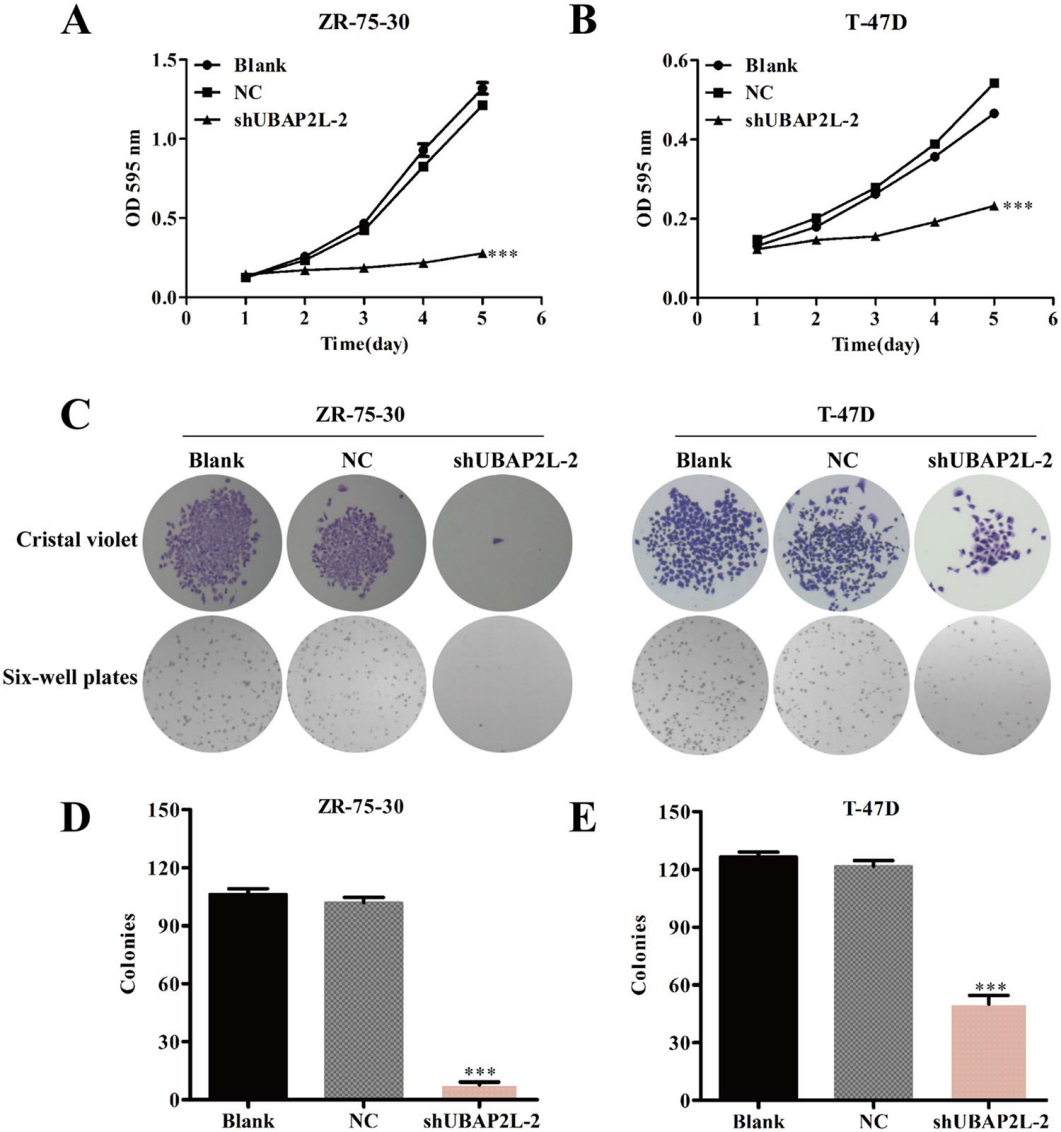
Silencing of UBAP2L down-regulated the proliferation and colony formation ability in breast cancer cells. **a**, **b** MTT assay showed significant differences in the proliferation of ZR-75-30 and T-47D cells between shUBAP2L-2 and blank or NC groups. **c** Representative light microscopic images regarding the size and number of colonies. **d**, **e** The number of colonies in shUBAP2L-2 group presented significant lower than the blank or NC group. Data represent the mean ± SD of triplicate samples. ***P < 0.001 vs. negative control (NC)

Cell proliferation was then determined on breast cancer cells after UBAP2L knockdown using MTT assay. As shown in Fig. 3a, b, growth curves in shUBAP2L-2 group were much lower than those in NC or blank groups in both ZR-75-30 and T-47D cells. On day 5, knockdown of UBAP2L reduced the proliferation rates of ZR-75-30 and T-47D cells by 83.3 and 53.6%, respectively, (*P* < 0.001). Furthermore, colony formation assay indicated that the size of single colony was visible smaller in shUBAP2L-2 group than that in NC or blank groups in ZR-75-30 (Fig. 3c) and T-47D cells (Fig. 3c). Statistical analysis (Fig. 3d, e) further demonstrated that the number of colonies was significantly reduced from NC group to shUBAP2L-2 group (ZR-75-30: 102 ± 12 vs. 8 ± 5, *P* < 0.001; T-47D: 122 ± 8 vs. 50 ± 12, *P* < 0.001). These results suggested that UBAP2L might play a positive role in malignant growth of breast cancer cells.

### Knockdown of UBAP2L induces G2/M phase arrest in breast cancer cells

**Fig. 4:**
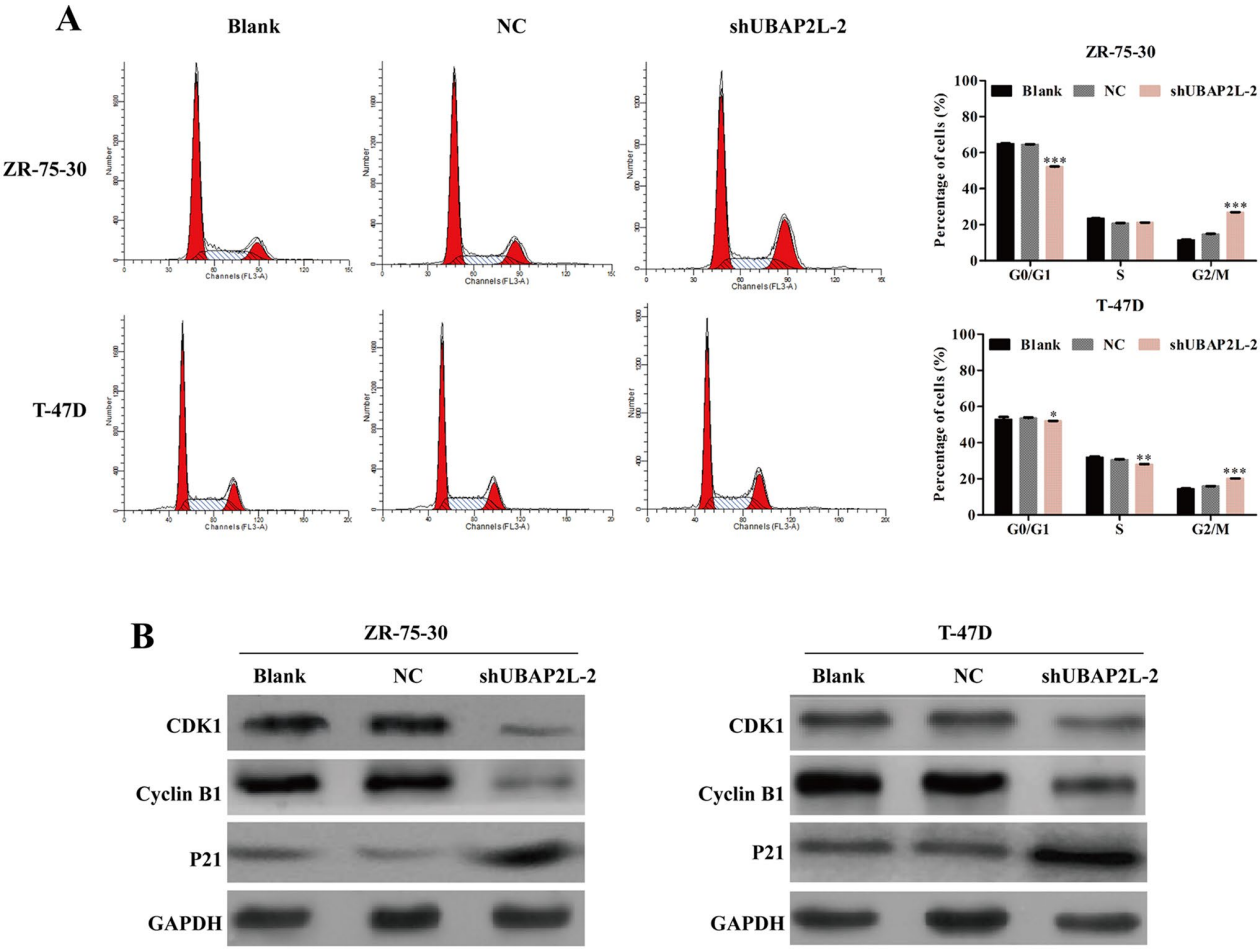
Silencing of UBAP2L induced G2/M phase arrest in breast cancer cells. **a** The percentage of cell cycle distribution was analyzed using flow cytometry. Statistical analysis showed that more cells were accumulated at G2/M phase in shUBAP2L-2 group as compared with the control group in ZR-75-30 and T-47D cells. **b** Differences in protein expression level of Cyclin B1, CDK1 and P21 between shUBAP2L-2 and NC or blank groups were determined by Western blot in ZR-75-30 and T-47D cells. Data represent the mean ± SD of triplicate samples. *P < 0.05, ***P < 0.01, ***P < 0.001 vs. negative control (NC)

To elucidate the potential mechanism underlying cell growth inhibition induced by UBAP2L silencing, cell cycle distribution was evaluated using flow cytometry. As shown in Fig. 4a, UBAP2L knockdown significantly increased the percentage of cells in G2/M phase in ZR-75-30 (14.71 ± 1.37 vs. 26.79 ± 1.48%, *P* < 0.001) and T-47D cells (15.91 ± 0.82 vs. 20.10 ± 0.48%, *P* < 0.001) and decreased the percentage of cells in phase in ZR-75-30 (64.49 ± 0.24 vs. 50.09 ± 0.61%, *P* < 0.001) and T-47D cells (53.58 ± 0.43 vs. 51.94 ± 0.72%, *P* < 0.05). Meanwhile, the number of S-phase T-47D cells treated with shUBAP2L-2 was also markedly decreased (30.51 ± 0.39 vs. 27.96 ± 0.53%, *P* < 0.01), suggesting that knockdown of UBAP2L might induce cell G2/M phase arrest.

Cell cycle regulators, including CDKs and Cyclins play an important role in cell cycle progression. We thus detected the expression alterations of CDK1 and Cyclin B1, associated with G2-M transition in ZR-75-30 and T-47D cells after UBAP2L knockdown using Western blot analysis. As shown in Fig. 4b, both CDK1 and Cyclin B1 protein expression were decreased in shUBAP2L-2 groups, whereas the expression levels of p21 was increased in ZR-75-30 and T-47D cells following shUBAP2L-2 transfection.

### Meta-analysis of UBAP2L expression in breast cancer vs. normal breast tissues using Oncomine microarray database

**Fig. 5:**
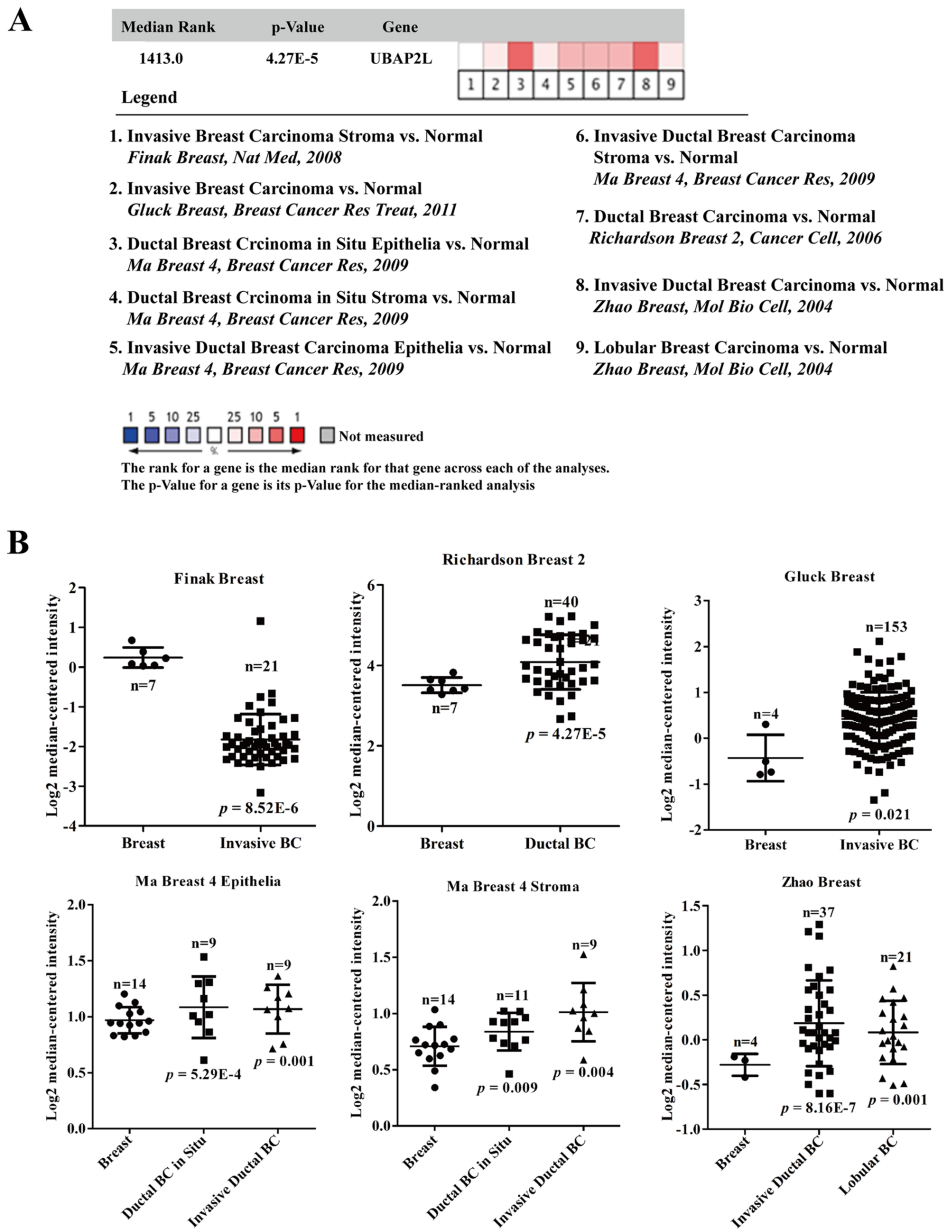
Oncomine microarray database was used to analyze UBAP2L mRNA expression in breast cancer vs. normal breast tissues. **a** Five microarray datasets regarding UBAP2L mRNA expression in breast cancer vs. normal tissues were included in our meta-analysis. Data are shown as the median rank of UBAP2L through each dataset analysis. *P* value for UBAP2L was presented using the median ranked analysis about breast cancer vs. normal tissues. **b** Differential expressions of UBAP2L mRNA expression in several common tissue subtypes were analyzed in these five datasets

To further confirm our findings that UBAP2L is overexpressed in breast cancer, public microarray datasets from Oncomine database were used to carry out meta-analysis of UBAP2L gene expression. Total of five online microarray datasets containing mRNA level test of cancer vs. normal tissue were included in meta-analysis. As shown in Fig. 5a, meta-analysis collectively revealed that increased UBAP2L mRNA expression was associated with breast cancer as compared with the normal breast tissue (gene median rank: 1413.0, *P* = 4.27E−5). Further analysis showed UBAP2L mRNA expression in breast cancer containing ductal breast cancer and invasive breast cancer exhibited a significant difference as compared with the normal breast tissue (*P* < 0.01) in Ma Breast 4 [18] and Zhao Breast [19] datasets.

## Discussion

UBAP2L is an involver of the ubiquitin–proteasome pathway which degrades most intracellular proteins [20]. Accumulating evidences have demonstrated that UBAP2L has been implicated in the growth and metastasis of various types of tumors. For example, UBAP2L is upregulated and its knockdown inhibits tumorigenesis and metastasis in colorectal cancer [12]. Knockdown of UBAP2L led to reduced growth of the malignant glioma and correlated with blockage of G0/G1 cell cycle progression [13]. However, the biological function of UBAP2L in breast cancer has been largely uncovered.

In the current study, UBAP2L was found to be significantly upregulated in breast cancer tissues and cell lines. Using Oncomine public database, total of five datasets were screened for comparison of the differentially expressed UBAP2L in breast cancer and normal tissues. Consistently, the mRNA expression of UBAP2L was found to be significantly upregulated in breast cancer tissues in these datasets, except for Finak Breast, which might be ascribed to different specimen collection, sample size and inclusion criteria. Upregulated UBAP2L was further confirmed by meta-analysis carried out on these five datasets. In function analysis, downregulation of UBAP2L resulted in inhibition of proliferation and colony formation, and correlated with G2/M phase arrest. What’s more, depletion of UBAP2L leads to a decreased cell cycle associated proteins CDK1 and Cyclin B1 and an elevated CDK inhibitor protein p21. These results indicate that UBAP2L might serve as an oncogene in breast cancer cells and may greatly facilitate to the proliferation and cell cycle progression.

**Fig. 6:**
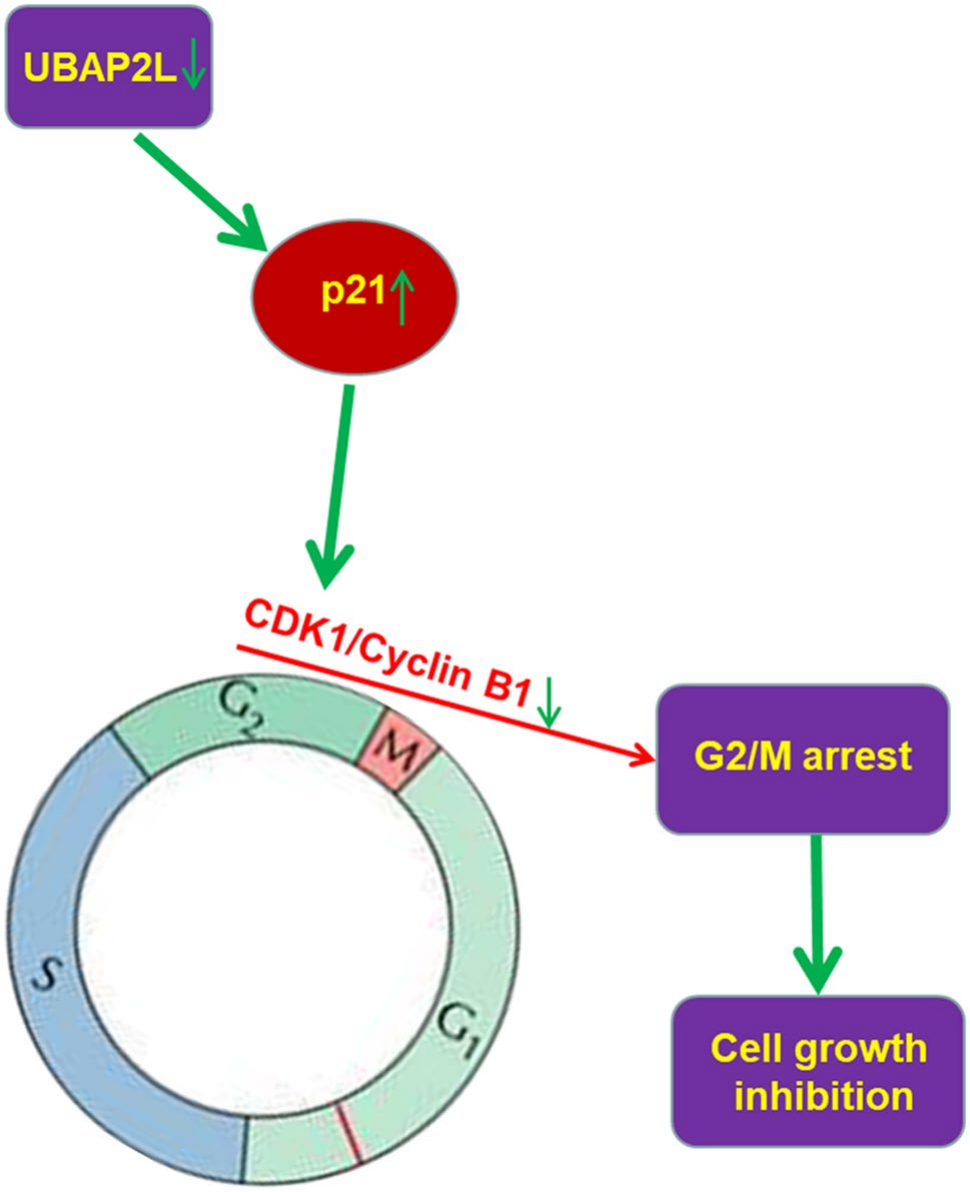
Schematic image on the speculation on the molecular relationship among UBAP2L, CDK1/Cyclin B1 and p21 in breast cancer cells

In eukaryotes, the decreased CDK1/Cyclin B1 complex formation correlates with G2/M phase accumulation [21]. P21, a mitosis regulator, can block the activation of CDK1 though binding to the CDK1/Cyclin B1 complex and arrest cells at G2/M phase [22]. The CDK–Cyclin inhibitory domain is located at the N terminus of p21, and the effectiveness is determined by the UPS [23]. Wilde et al. [9] verified the co-fractionation of UBAP2L with ubiquitin and presents high-density fraction, suggesting a component of UPS. In the present study, depletion of UBAP2L resulted in G2/M arrest, decreased CDK1 and Cyclin B1 protein expressions and corresponding increased P21 protein levels. We suppose that the decreased UBAP2L might disrupt the UPS to affect the effectiveness of the CDK–Cyclin inhibitory domain in the p21-N terminus, leading to G2/M phase arrest due to attenuated activation of the CDK1/Cyclin B1 complex (Fig. 6). Our results demonstrated that knockdown of UBAP2L inhibited breast cancer cells growth and proliferation, which might be ascribed to G2/M cell cycle arrest.

## Conclusion

In summary, our results suggest that UBAP2L plays a critical role for the development of breast cancer. UBAP2L knockdown impedes the breast cancer cell proliferation and colony formation might thought G2/M cell cycle arrest. The role of UBAP2L in cancer biology will supply a potential therapeutic target for breast cancer.
